# Social distancing patterns in nine municipalities of Rio Grande do Sul, Brazil: the Epicovid19/RS study

**DOI:** 10.11606/s1518-8787.2020054002810

**Published:** 2020-07-21

**Authors:** Aluisio J D Barros, Cesar G Victora, Ana M B Menezes, Bernardo L Horta, Fernando Hartwig, Gabriel Victora, Lúcia C Pellanda, Odir A Dellagostin, Claudio J Struchiner, Marcelo N Burattini, Marcelo R Gonçalves, Lia G Possuelo, Liliana P Weber, Sonara Lucia Estima, Nadège Jacques, Jenifer Härter, Shana G Silva, Matias Frizzo, Rosangela C Lima, Fernando C Barros, Mariângela F Silveira, Pedro C Hallal

**Affiliations:** I Universidade Federal de Pelotas Centro de Pesquisas Epidemiológicas PelotasRS Brasil Universidade Federal de Pelotas. Centro de Pesquisas Epidemiológicas. Pelotas, RS, Brasil; II The Rockefeller University New YorkNY USA The Rockefeller University. New York, NY, USA; III Universidade Federal de Ciências de Saúde de Porto Alegre Porto AlegreRS Brasil Fundação Universidade Federal de Ciências de Saúde de Porto Alegre. Porto Alegre, RS, Brasil; IV Universidade Federal de Pelotas Centro de Desenvolvimento Tecnológico PelotasRS Brasil Universidade Federal de Pelotas. Centro de Desenvolvimento Tecnológico. Pelotas, RS, Brasil; V Fundação Getúlio Vargas Escola de Matemática Aplicada Rio de JaneiroRJ Brasil Fundação Getúlio Vargas. Escola de Matemática Aplicada. Rio de Janeiro, RJ, Brasil; VI Universidade de São Paulo Faculdade de Medicina São PauloSP Brasil Universidade de São Paulo. Faculdade de Medicina. São Paulo, SP, Brasil; VII Universidade Federal de São Paulo Hospital São Paulo São PauloSP Brasil Universidade Federal de São Paulo. Hospital São Paulo. São Paulo, SP, Brasil; VIII Universidade Federal do Rio Grande do Sul Porto Alegre,RS Brasil Universidade Federal do Rio Grande do Sul. Porto Alegre, RS, Brasil; IX Universidade de Santa Cruz do Sul Santa Cruz do SulRS Brasil Universidade de Santa Cruz do Sul. Santa Cruz do Sul, RS, Brasil; X Universidade de Caxias do Sul Caxias do SulRS Brasil Universidade de Caxias do Sul. Caxias do Sul, RS, Brasil; XI Universidade La Salle CanoasRS Brasil Universidade La Salle (Unilasalle). Canoas, RS, Brasil; XII Universidade Federal do Pampa UruguaianaRS Brasil Universidade Federal do Pampa. Uruguaiana, RS, Brasil; XIII Universidade Federal da Fronteira Sul Passo FundoRS Brasil Universidade Federal da Fronteira Sul. Passo Fundo, RS, Brasil; XIV Universidade Regional do Noroeste do Estado do Rio Grande do Sul IjuíRS Brasil Universidade Regional do Noroeste do Estado do Rio Grande do Sul (UNIJUÍ). Ijuí, RS, Brasil; XV Universidade Federal de Santa Maria Santa MariaRS Brasil Universidade Federal de Santa Maria. Santa Maria, RS, Brasil; XVI Universidade Católica de Pelotas Faculdade de Medicina PelotasRS Brasil Universidade Católica de Pelotas. Faculdade de Medicina. Pelotas, RS, Brasil

**Keywords:** Coronavirus Infections, prevention & control, Health Knowledge, Attitudes, Practice, Health Risk Behaviors, Socioeconomic Factors

## Abstract

**OBJECTIVE:**

To describe social distancing practices in nine municipalities of the state of Rio Grande do Sul, Brazil, stratified by gender, age, and educational attainment.

**METHODS:**

Two sequential cross-sectional studies were conducted in the municipalities of Canoas, Caxias do Sul, Ijuí, Passo Fundo, Pelotas, Porto Alegre, Santa Cruz do Sul, Santa Maria, and Uruguaiana to estimate the population prevalence of COVID-19. The study was designed to be representative of the urban population of these municipalities. A questionnaire including three questions about social distancing was also administered to the participants. Here, we present descriptive analyses of social distancing practices by subgroups and use chi-square tests for comparisons.

**RESULTS:**

In terms of degree of social distancing, 25.8% of the interviewees reported being essentially isolated and 41.1% reported being quite isolated. 20.1% of respondents reported staying at home all the time, while 44.5% left only for essential activities. More than half of households reported receiving no visits from non-residents. Adults aged 20 to 59 reported the least social distancing, while more than 80% of participants aged 60 years or older reported being essentially isolated or quite isolated. Women reported more stringent distancing than men. Groups with higher educational attainment reported going out for daily activities more frequently.

**CONCLUSIONS:**

The extremes of age are more protected by social distancing, but some groups remain highly exposed. This can be an important limiting factor in controlling progression of the COVID-19 pandemic.

## INTRODUCTION

Since the World Health Organization characterized the 2019 coronavirus disease (Covid-19) outbreak as a pandemic on March 11, 2020, states and municipalities across Brazil have begun to adopt social distancing policies and strategies, with the support of the Ministry of Health. Despite slightly different emphases and strategies, most of the country quickly adopted measures to restrict personal contact—so-called social distancing, including advice to stay at home, school closures, bans on activities and venues that cause crowding (such as sports events and shopping malls), and constraints on the operation of commercial establishments. This generally meant closing most retail establishments, except supermarkets, grocery stores, drugstores/pharmacies, and other essential facilities^1^.

Since the start of the pandemic, there has been mounting evidence that social distancing can reduce the spread of SARS-CoV-2. A study in Hong Kong found a 44% reduction in effective reproduction number (R_t_) after the implementation of social distancing measures, particularly school closures^2^. A meta-analysis of 29 studies (25 of which were modeling studies) also concluded that social distancing measures can check the spread of Covid-19, especially when combined with broader restrictions, such as school closures and travel bans^3^. Another meta-analysis studying the effects of distancing and the use of masks and eye protection showed that physical distancing reduces the risk of infection by approximately 80% (relative risk, 95% CI: 0.10-0.41). The effective distance was estimated at >1 m (preferably 2 m). Mask wearing has also proven highly effective^4^.

In Brazil, the effect of social distancing on the spread of the epidemic has been evaluated in three studies using data from In Loco, a company which provides intelligence based on location data^a^. One of these studies found an inverse association between social distancing and Covid-19 spread, as well as a positive association between air mobility and spread. Climate and socioeconomic characteristics were only weakly associated^5^. Another study found a strong negative correlation (r < –0.7) between the proportion of people staying at home and R_t_^6^. The “social isolation index” calculated by In Loco was also incorporated into an elasticity model which showed that, on average, every 10% increase in the isolation index was associated with 26% fewer cases of Covid-19 and 18% fewer deaths^7^.

Nevertheless, sources of mobility data, such as In Loco and Google^b^, are unable to characterize subgroups of community populations. One cannot tell from these data whether those staying at home are younger or older adults, men or women. Thus, in the present investigation, we use data from the Epicovid19/RS study^c^, designed to estimate the population prevalence of SARS-CoV-2 infection in the Brazilian state of Rio Grande do Sul, to present social distancing patterns in nine surveyed municipalities, assessing differences by city, age, sex, and educational attainment.

## METHODS

The Epicovid19/RS study is being carried out in nine sentinel municipalities across the state of Rio Grande do Sul. These municipalities—Canoas, Caxias do Sul, Ijuí, Passo Fundo, Pelotas, Porto Alegre, Santa Cruz do Sul, Santa Maria, and Uruguaiana—were chosen because they are the largest of each of the state’s geographic mesoregions, as defined by the Brazilian Institute of Geography and Statistics (IBGE), plus the second-largest municipality in the Greater Porto Alegre area.

In each of the municipalities, a sample of 500 households was selected by drawing of 50 urban census tracts with probability proportional to size and 10 households per tract. The households were selected randomly during the first round of the study from a list of addresses provided by IBGE. For the second round, households were selected systematically by skipping to the 10th household over from each of the households surveyed in the previous round. Households where no one was present at the time of the interview, or whose residents refused to participate, were replaced by the neighboring residence. In each selected household, a list of residents was compiled, and one resident was selected at random to be interviewed and tested for Covid-19. The study protocol provides for four independent rounds to be conducted, one every 2 weeks. The first and second rounds took place between 11–13 and 25–27 April, 2020, respectively. Testing was performed with a rapid serology assay that tests for the presence of anti-SARS-CoV-2 IgM and IgG and yields a result within 15 minutes. This test was previously validated by our group^8^. More detailed information on methodology has been published elsewhere^9^.

The study questionnaire was designed to collect information on sex, age, educational attainment of the respondent, highest educational attainment within the household, social distancing practices, co-morbidities, and symptoms of Covid-19. The three questions on social distancing are of particular interest to the present analysis. The first item asked, “To what extent do you are managing to follow the social distancing guidance from the health authorities, i.e., staying at home and avoiding contact with others?”. This was scored on a five-point scale, with alternatives read aloud to the respondent: very little; little; some; quite; and practically isolated from everyone. The second question was, “What have your routine activities been?”. The alternatives were: staying home all the time; only leaving home only for essentials, such as groceries; leaving home from time to time to run errands and stretch legs; going out every day for regular activities; and out of the house all day, every day, either for work or for other regular activities. Finally, respondents were asked “Who has been in the house?”. The alternatives were: only those relatives who also live in the house, if any—no one else; some close relatives visit once or twice a week; some close relatives visit nearly every day; friends, distant relatives, or others visit once or twice a week; and friends, distant relatives, or others visit nearly every day. If the randomly selected respondent was a child (under age 12) or an older adult who was unable to answer, the question was asked of the respondent’s legal guardian.

Field work was carried out by Instituto Pesquisas de Opinião^d^, a contract research organization, with the aid of universities in each of the selected municipalities. Interviewers were selected among students of health programs at partner universities. All were trained in performing the rapid test and in administering the questionnaire. The questionnaire itself is included at the end of the supplementary material. All relevant biological safety guidance was followed to protect interviewers and respondents alike.

The study protocol was approved by the National Research Ethics Committee (CONEP, opinion number: 30721520.7.1001.5313). All respondents, or their legal guardians in case of children under 12 and disabled older adults, provided written informed consent for participation after receiving information about the objectives and procedures of the study.

The statistical analyses presented herein are essentially descriptive and were based on group percentages and bar charts. When necessary, frequencies were compared using chi-square tests.

## RESULTS

The numbers of interviews carried out during the first two rounds of the Epicovid19/RS study, on April 11–13 and April 25–27, are shown in Table 1 (overall and by municipality). It also shows the distribution of the sample by sex, age, and educational level. A chi-square test was used to compare municipalities. There was a female predominance in the sample, with women accounting for nearly 60 of the 8,611 interviews conducted. We also found that the age distribution differed significantly from the population distribution estimated for 2020 by IBGE.^e^ Overall, 12%, 13%, 30%, 26%, and 19% of respondents were aged 0–10, 11–19, 20–39, 40–59, and 60+ years. There were thus far fewer children and adolescents and far more older adults in our sample than would be expected in the population. This is probably attributable to school-aged residents being absent from home at the time of contact, as well as refusals to take the rapid test, which involves a fingerstick blood draw. Among all households approached, 9% refused to participate and 11% were replaced, the vast majority because there was no one home.

**Table 1:** Sample distribution by municipality of residence, sex, age, and educational attainment for rounds 1 and 2. Epicovid19/RS study, April 2020.

	Canoas	Caxias do Sul	Ijuí	Passo Fundo	Pelotas	Porto Alegre	Santa Cruz do Sul	Santa Maria	Uruguaiana	Overall
Total N	832	1,000	923	1,000	1,000	896	1,000	961	999	8,611
Sex (p = 0.112)										
Male	45.2	43.2	39.7	41.3	39.5	39.8	42.5	39.8	39.2	41.1
Female	54.8	56.8	60.4	58.7	60.5	60.2	57.5	60.3	60.8	58.9
Age (p < 0.001)										
0-10	1.8	3.4	3.5	3.1	4.0	2.5	3.9	2.9	4.6	3.3
11-19	2.8	6.3	4.9	5.4	4.5	2.7	5.6	5.1	6.2	4.9
20-39	30.4	26.4	28.0	32.0	28.1	26.3	26.7	30.9	23.9	28.1
40-59	33.5	34.7	30.6	30.8	32.8	34.9	33.3	31.8	33.2	32.9
60+	31.5	29.1	33.1	28.7	30.6	33.6	30.5	29.2	32.0	30.9
Educational attainment of respondent (p < 0.001)										
Primary (0-4 years of schooling)	5.7	5.5	7.5	3.4	7.0	3.3	6.5	4.1	5.1	5.3
Primary (5+ years of schooling)	25.6	29.7	33.3	27.9	29.6	18.7	34.1	23.0	34.8	28.7
Secondary	33.8	31.9	31.2	31.3	31.5	29.8	28.9	31.7	35.6	31.7
Some higher education	12.8	12.3	8.7	9.8	9.6	11.1	9.8	11.0	7.1	10.2
Higher (undergraduate or graduate) degree	22.2	20.7	19.3	27.6	22.4	37.0	20.7	30.3	17.3	24.1

Note: P-values refer to comparisons between municipalities by a chi-square test.

The most frequent response to the question “To what extent are you socially distancing” was “quite”, accounting for 41.1% of answers (Table 2). The least frequent answer was “very little” (5.8%). Overall, 25.8% of respondents claimed they had been practically isolated at home.

**Table 2:** Distribution of the three selected social distance indicators for rounds 1 and 2. Epicovid19/RS study, April 2020.

	N	%	95% CI	
To what extent are you socially distancing?				
Isolated	2,225	25.8	24.6	27.1
Quite	3,538	41.1	39.8	42.3
Some	1,698	19.7	18.8	20.7
Little	648	7.5	6.9	8.2
Very little	502	5.8	5.3	6.4
What have your routine activities been?				
I have been staying home all the time	1,727	20.1	19.1	21.1
I have only been leaving home only for essentials, such as groceries	3,836	44.5	43.3	45.9
I have been leaving home from time to time to run errands and stretch my legs	894	10.4	9.6	11.2
I have been going out every day for regular activities	485	5.6	5.1	6.2
I have been out of the house all day, every day, either for work or for other regular activities	1,669	19.4	18.3	20.5
Who has been in the house?				
Only those relatives who also live in the house, if any—no one else	4,584	53.2	51.6	54.9
Close relatives visit once or twice a week	2,583	30.0	28.7	31.3
Close relatives visit nearly every day	631	7.3	6.6	8.1
Distant relatives or other people visit once or twice a week	458	5.3	4.8	5.9
Distant relatives or other people visit nearly every day	355	4.1	3.6	4.7

The majority claimed to go out only for essential activities (44.5%), while 20.1% reported staying at home all the time. However, 19.4% of respondents stated they left the house every day (Table 2). To better understand this group, we will explore its characteristics further. Among those who reported leaving the house every day for work or regular activity, there was a significant predominance of men (54.9%, p < 0.001) and adults (90.3% were aged 20–59, p < 0.001). Only 7.6% of those who claimed to leave the house every day were aged 60 or over, versus 36.5% of those who do not go out every day. Among those who reported going out every day, there was also a predominance of respondents with a higher education (p < 0.001): 39.2% of those who leave the house every day have some higher education or a higher (undergraduate or graduate) degree, versus 33.1% of those who do not go out every day. These results are presented in Table 3.

**Table 3:** Profile of respondents who reported being out of the house all day, every day, for regular activities. Epicovid19/RS study, April 2020.

	Leaves house every day	

	Yes (%)	No (%)
Sex (n=8,611)		
Male	54.9	37.8
Female	45.1	62.2
Age (n=8,609)		
0-10	0.3	4.1
11-19	1.8	5.6
20-39	47.1	23.5
40-59	43.2	30.4
60+	7.6	36.5
(N= 8,350)		
Primary (0-4 years of schooling)	2.8	6.0
Primary (5+ years of schooling)	18.6	31.2
Secondary	39.4	29.8
Incomplete higher education	12.3	9.7
Higher (undergraduate or graduate) degree	26.9	23.4

Regarding visitation, more than half of the interviewees reported not letting anyone other than residents themselves in the house. Less than 10% reported visits by non-family members.

**Figure 1:**
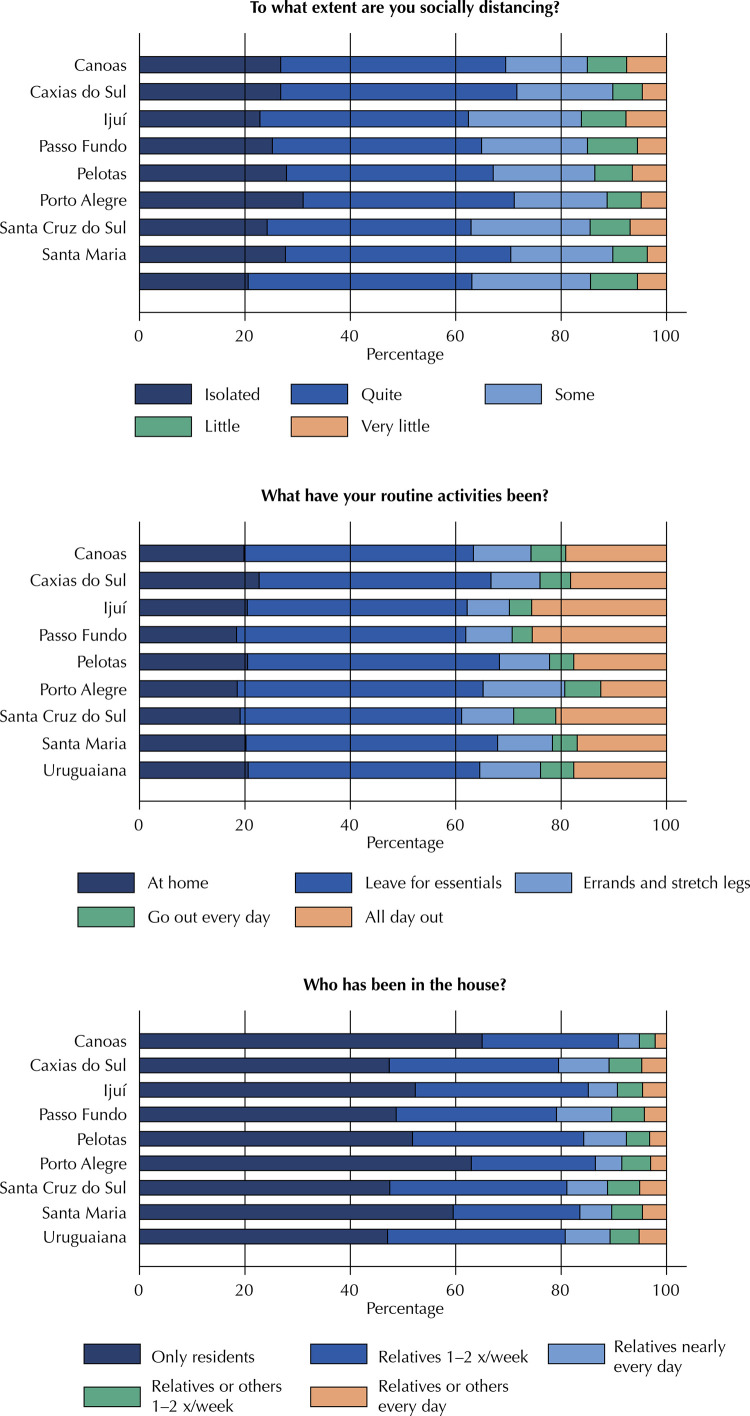
Social distancing indicators by municipality surveyed, using data from rounds 1 and 2. P-values for degree of distancing, routine activities, and visitation, all p < 0.001. Epicovid19/RS study, April 2020.

**Figure 2:**
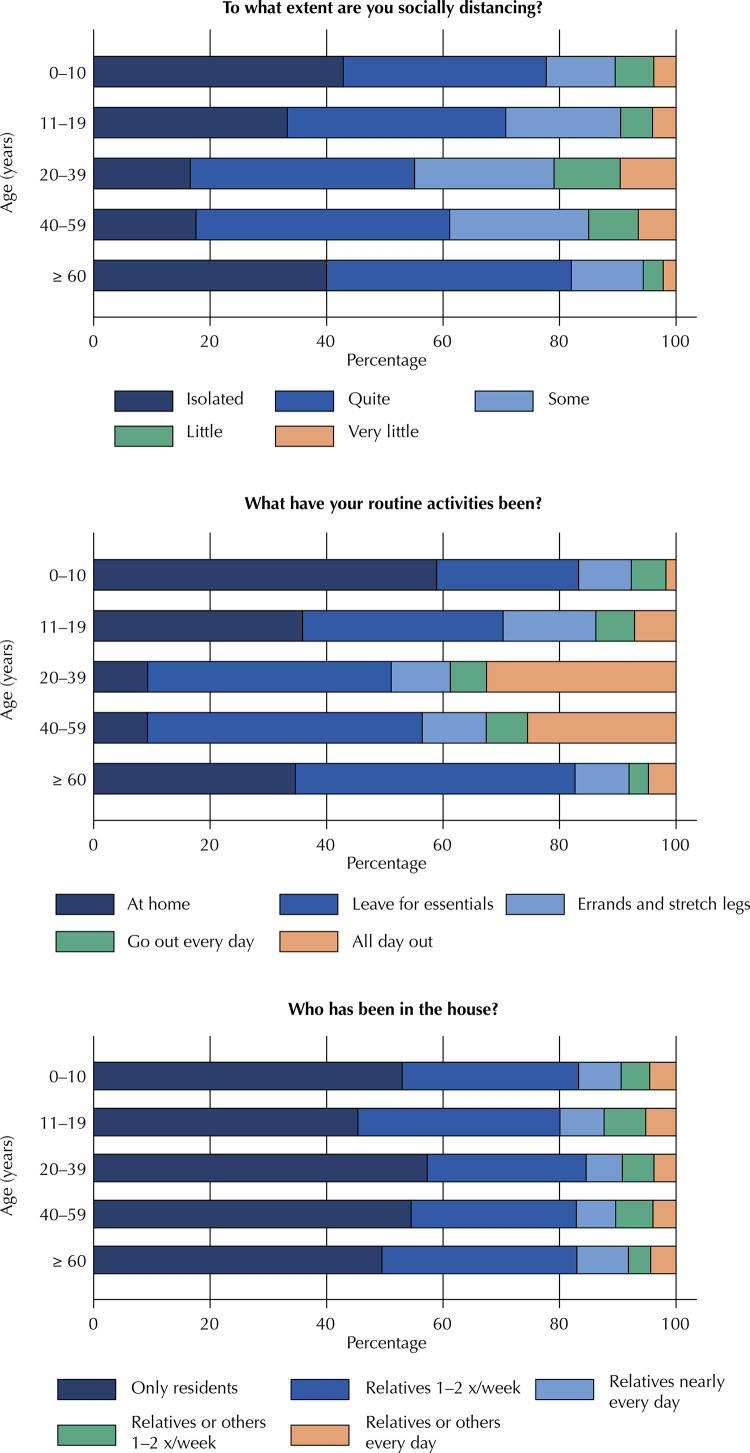
Social distancing indicators by respondent age range, using data from rounds 1 and 2. P-values for degree of distancing, routine activities, and visitation, all p < 0.001. Epicovid19/RS study, April 2020.

**Figure 3:**
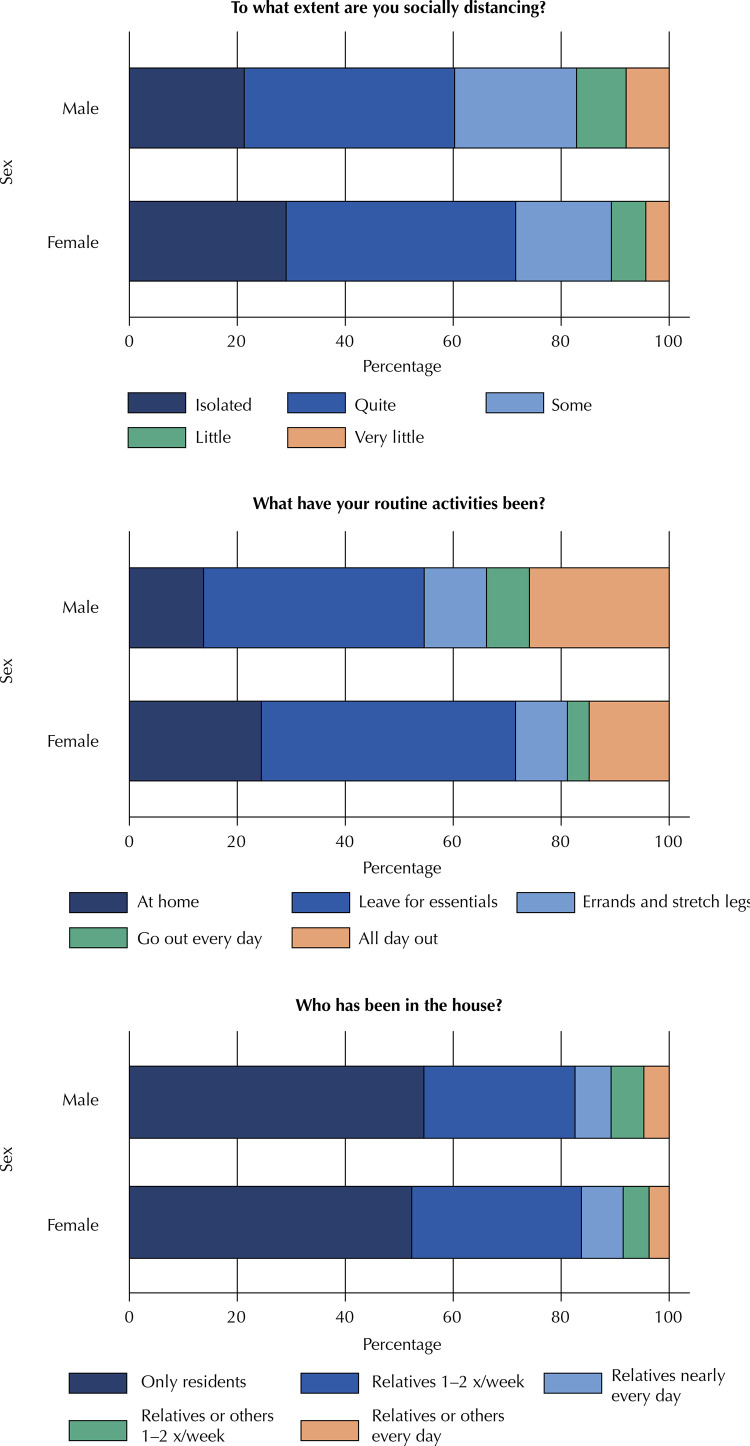
Social distancing indicators by respondent sex, using data from rounds 1 and 2. P-values for degree of distancing, routine activities, and visitation, all p < 0.001. Epicovid19/RS study, April 2020.

**Figure 4:**
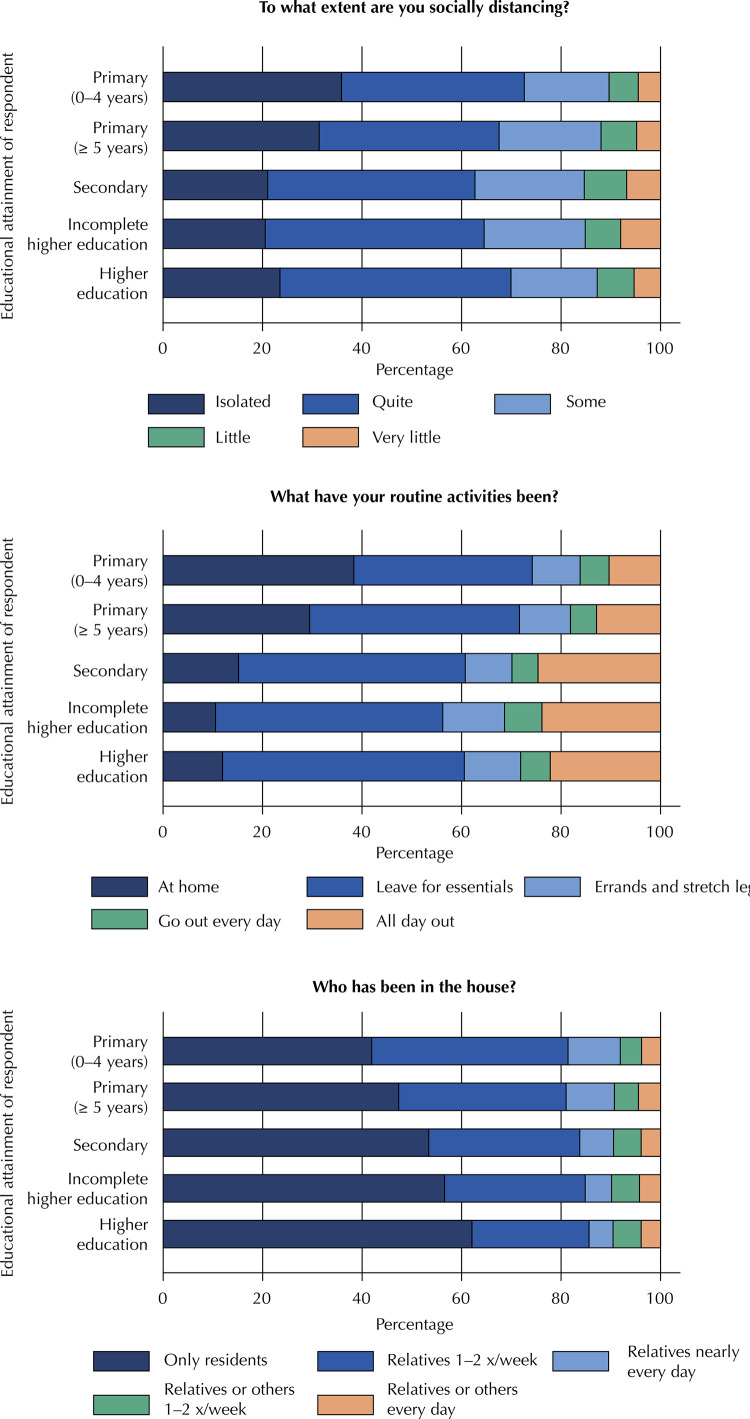
Social distancing indicators by respondent educational attainment, using data from rounds 1 and 2. P-values for degree of distancing, routine activities, and visitation, all p < 0.001. Epicovid19/RS study, April 2020.

Analysis of social distancing indicators by municipality, age, sex, and education revealed statistically significant differences in all cases. The results are shown in Figures 1 to 4 and in Tables 4 to 7 [Table t5] [Table t6] [Table t7]. Porto Alegre and Santa Maria exhibited the highest degree of social distancing, while Uruguaiana and Ijuí had a less favorable pattern. Regarding routine activities, there was no major difference across municipalities in the percentage of respondents who report staying home all day, but a strikingly higher percentage reported being “out of the house all day” in Ijuí and Passo Fundo. Canoas stands out in terms of movement in and out of households, with the highest proportion of residents alone being allowed in the house and the lowest proportion of relatives and non-relatives; closely followed by Porto Alegre and Santa Maria, also with favorable patterns.

**Table 4:** Social distancing indicators by participating municipality. Epicovid19/RS study, April 2020.

	Canoas	Caxias do Sul	Ijuí	Passo Fundo	Pelotas	Porto Alegre	Santa Cruz do Sul	Santa Maria, RS	Uruguaiana
To what extent are you socially distancing? (p < 0.001)									
Isolated	26.8	26.8	22.9	25.2	27.9	31.0	24.2	27.7	20.6
Quite	42.7	44.8	39.5	39.7	39.2	40.1	38.7	42.8	42.4
Some	15.5	18.2	21.5	20.1	19.3	17.6	22.6	19.4	22.5
Little	7.5	5.6	8.5	9.5	7.1	6.5	7.6	6.6	8.9
Very little	7.6	4.6	7.7	5.5	6.5	4.8	6.9	3.6	5.5
What have your routine activities been? (p < 0.001)									
I have been staying home all the time	19.8	22.7	20.5	18.4	20.5	18.5	19.1	20.2	20.6
I have only been leaving home only for essentials, such as groceries	43.5	44.0	41.7	43.5	47.8	46.7	42	47.8	43.9
I have been leaving home from time to time to run errands and stretch my legs	10.9	9.3	8.0	8.8	9.5	15.5	9.9	10.4	11.5
I have been going out every day for regular activities	6.6	5.8	4.2	3.8	4.6	6.8	8.0	4.7	6.3
I have been out of the house all day, every day, either for work or for other regular activities	19.1	18.2	25.6	25.5	17.6	12.5	21	17.0	17.6
Who has been in the house? (p < 0.001)									
Only those relatives who also live in the house, if any—no one else	65.0	47.4	52.3	48.7	51.8	63.0	47.5	59.5	47.1
Close relatives visit once or twice a week	25.8	32.1	32.8	30.4	32.5	23.6	33.6	24.0	33.7
Close relatives visit nearly every day	4.0	9.6	5.5	10.5	8.1	5.0	7.7	6.0	8.5
Distant relatives or other people visit once or twice a week	3.0	6.2	4.8	6.2	4.4	5.5	6.1	5.8	5.5
Distant relatives or other people visit nearly every day	2.2	4.7	4.6	4.2	3.2	3.0	5.1	4.6	5.2

**Table 5 t5:** Social distancing indicators by age group. Epicovid19/RS study, April 2020.

	Age (years)				

	0-10	11-19	20-39	40-59	60+
To what extent are you socially distancing? (p < 0.001)					
Isolated	42.9	33.3	16.6	17.6	40.0
Quite	34.8	37.5	38.5	43.6	42.0
Some	11.9	19.7	23.9	23.9	12.3
Little	6.6	5.5	11.4	8.5	3.4
Very little	3.8	4.0	9.6	6.5	2.2
What have your routine activities been? (p < 0.001)					
I have been staying home all the time	58.9	35.9	9.3	9.3	34.7
I have only been leaving home only for essentials, such as groceries	24.4	34.4	41.8	47.2	48.0
I have been leaving home from time to time to run errands and stretch my legs	9.1	15.9	10.1	11.0	9.3
I have been going out every day for regular activities	5.9	6.7	6.2	7.1	3.4
I have been out of the house all day, every day, either for work or for other regular activities	1.7	7.1	32.6	25.5	4.7
Who has been in the house? (p < 0.001)					
Only those relatives who also live in the house, if any—no one else	53.0	45.4	57.3	54.5	49.5
Close relatives visit once or twice a week	30.3	34.7	27.3	28.3	33.5
Close relatives visit nearly every day	7.32	7.6	6.2	6.8	8.9
Distant relatives or other people visit once or twice a week	4.9	7.1	5.4	6.4	3.8
Distant relatives or other people visit nearly every day	4.5	5.2	3.8	4.0	4.4

**Table 6 t6:** Social distancing indicators by sex of respondent. Epicovid19/RS study, April 2020.

	Sex	

	Male	Female
To what extent are you socially distancing? (p < 0.001)		
Isolated	21.3	29.0
Quite	39.0	42.5
Some	22.6	17.7
Little	9.2	6.4
Very little	8.0	4.3
What have your routine activities been? (p < 0.001)		
I have been staying home all the time	13.7	24.5
I have only been leaving home only for essentials, such as groceries	40.9	47.1
I have been leaving home from time to time to run errands and stretch my legs	11.5	9.6
I have been going out every day for regular activities	8.0	4.0
I have been out of the house all day, every day, either for work or for other regular activities	25.9	14.8
Who has been in the house? (p < 0.001)		
Only those relatives who also live in the house, if any —no one else	54.6	52.3
Close relatives visit once or twice a week	28.0	31.4
Close relatives visit nearly every day	6.7	7.8
Distant relatives or other people visit once or twice a week	6.1	4.8
Distant relatives or other people visit nearly every day	4.7	3.7

**Table 7 t7:** Social distancing indicators by educational attainment of respondent. Epicovid19/RS study, April 2020.

	Educational attainment				

	Primary (0–4 years)	Primary (5+ years)	Secondary	Some higher education	Higher degree
To what extent are you socially distancing? (p < 0.001)					
Isolated	35.9	36.8	17.0	5.8	4.5
Quite	31.4	36.2	20.5	7.1	4.8
Some	21.0	41.7	22.0	8.5	6.8
Little	20.5	44.0	20.3	7.2	8.0
Very little	23.5	46.5	17.3	7.4	5.3
What have your routine activities been? (p < 0.001)					
I have been staying home all the time	38.3	35.9	9.6	5.8	10.3
I have only been leaving home only for essentials, such as groceries	29.5	42.2	10.3	5.2	12.9
I have been leaving home from time to time to run errands and stretch my legs	15.1	45.6	9.4	5.3	24.6
I have been going out every day for regular activities	10.6	45.7	12.4	7.5	23.8
I have been out of the house all day, every day, either for work or for other regular activities	11.9	48.6	11.3	6.0	22.1
Who has been in the house? (p < 0.001)					
Only those relatives who also live in the house, if any —no one else	41.9	39.5	10.5	4.3	3.8
Close relatives visit once or twice a week	47.4	33.7	9.7	4.9	4.4
Close relatives visit nearly every day	53.4	30.4	6.8	5.6	3.9
Distant relatives or other people visit once or twice a week	56.6	28.3	5.3	5.6	4.2
Distant relatives or other people visit nearly every day	62.1	23.5	4.8	5.7	3.9

Social distancing and routine activities follow a very clear U-shaped pattern in relation to age (Figure 2). Respondents aged 20 to 59 were those least likely to report being practically isolated or staying at home all day. There is a very large concentration of “going out all day, every day” responses in this age group. The 60-and-older age group appears to be very well protected, with more than 80% reporting near-isolation or a high level of social distancing, either staying home all day or leaving the house only for bare essentials. Movement in and out of the house follows a less clear pattern in relation to the respondent’s age, which is to be expected, since visitation practices are much less dependent on a single resident than on the household as a whole.

Regarding sex (Figure 3), women were clearly more able to maintain social distancing and restrict routine activities, with a greater proportion of women than men reporting near-isolation and staying home all the time. Movement in and out of the house differed little by sex, despite statistical significance, as observed for age.

The respondent’s educational attainment had a clear association with social distancing and routine activities (Figure 4). Assessment of education in the group that reported being practically isolated or “quite” able to maintain social distancing again revealed a U-shaped pattern, although not as striking as for age. Individuals with a secondary-level or some higher education (but no degree) were those who least adhered to social distancing. Conversely, regarding routine activities, individuals with a higher degree tended to be those who least reported staying home all the time. At the other end of the scale, respondents with a secondary education or higher were the ones who most often reported going out for work on a daily basis. Restricting these analyses to adults and the elderly (to remove individuals outside the potentially employed age group) did not change the observed patterns at all. Finally, we found a clear association between the percentage of households admitting family members alone and higher education. In other words, individuals with a higher educational level were more likely to leave the house but were far more restrictive when it came to allowing non-family members to visit.

## DISCUSSION

This study provides an overview of social distancing patterns in 9 municipalities across the Brazilian state of Rio Grande do Sul, stratified by sociodemographic characteristics. The context of the state is one of low prevalence of SARS-CoV-2 exposure, as shown by the first two rounds of Epicovid19/RS, with point prevalence estimates of 0.05% and 0.13% respectively^10^. Between the first and the second rounds of the study, a statewide process of gradual lifting of restrictions on some business sectors began, the effect of which on social distancing indicators should be more clearly noticeable after the third round of surveys. From the point of view of distancing patterns, there was no relevant change from the first to the second round; thus, we believe their data are best presented jointly.

Information on social distancing and mobility is based on reporting and may be subject to recall bias or, even more importantly, social expectation bias. In the current scenario, respondents may be embarrassed to reveal low compliance with recommended distancing practices. Our results may thus overestimate social distancing in the sample. Another limitation is that the sample was not constructed in a manner representative of the state, as it was based on a deliberately selected subset of municipalities. Nevertheless, we believe the picture presented herein gives an informative and useful profile of population behavior in the surveyed municipalities, which account for a significant portion of the state population.

In brief, 65% of respondents are following social distancing guidelines and more than 80% of households have restricted visits to residents or close relatives, up to 2 times a week. However, there were quite significant variations between municipalities. Two of the three municipalities with the highest percentage of households in which visitation was restricted to family and residents are in the Greater Porto Alegre area, but the third is a smaller town located in the center of the state. There is no clearly identifiable pattern related to city size or geographic location.

Regarding age and education, our findings show that children, adolescents, and older adults are most protected in terms of social distancing, with adults aged 20 to 59 being the most exposed group. This is an important finding, given the consensus in the literature that the elderly and persons with comorbidities are most at risk for severe Covid-19 and death^11,12^.

The more educated segment of the population appears to be less protected in our analysis. When we specifically analyzed the group that reports leaving home every day for regular activities, it was essentially composed of predominantly adult, university-educated males. This finding is quite surprising, as we expected this profile of professionals to be working remotely from home, with blue-collar workers—particularly in the construction industry and the trades—would instead be leaving for work on a daily basis. Perhaps the drastic reduction in economic activities may explain this finding. On the other hand, more educated households also reported less movement of non-residents in and out of the home.

In conclusion, we found that social distancing patterns varied significantly across the surveyed municipalities and among population subgroups with different sociodemographic characteristics. Older adults, one of the main high-risk groups, exhibited better social distancing behavior. The same was true of children and adolescents. Adults with a higher level of education exhibited the lowest adherence to social distancing recommendations.

Completion of subsequent rounds of the Epicovid19/RS study should allow us to assess changes in behavior in response to the easing of lockdown measures since late April 2020.

## Footnotes

a Inloco. Enfrentando a COVID19. São Paulo; c2020 [cited 2020 May 26]. Available from: http://www.inloco.com.br/covid-19

b Google. COVID-19: relatórios de mobilidade da comunidade. [cited 2020 May 26]. Available from: https://www.google.com/covid19/mobility/

c Hallal PC, Horta BL, Barros AJD, Dellagostin AO, Hartwig FP, Pellanda LC, et al. Epidemiologia da COVID19 no Rio Grande do Sul: estudo de base populacional. Pelotas, RS: EPICOVID19; c2020 [cited 2020 May 26]. Available from: http://www.rs.epicovid19brasil.org

d Instituto Pesquisas de Opinião. Porto Alegre, RS: IPO; c2016 [cited 2020 May 26]. Available from: https://www.ipo.inf.br/

e Instituto Brasileiro de Geografia e Estatística. Projeções da População. Rio de Janeiro: IBGE; [cited 2020 May 26]. Available from: https://www.ibge.gov.br/estatisticas/sociais/populacao/9109-projecao-da-populacao.html?=&t=resultados
