# Supplementary material for: Social distancing patterns in nine municipalities of Rio Grande do Sul, Brazil: the Epicovid19/RS study
Source: Rev Saude Publica. 2020 Jul 21;54:75. doi: 10.11606/s1518-8787.2020054002810 (PMC7373222; doi:10.11606/s1518-8787.2020054002810)
Supplement: Translation

# Translation: Padrões de distanciamento social em nove cidades gaúchas: estudo Epicovid19/RS

**Authors:** Aluisio J D Barros, Cesar G Victora, Ana M B Menezes, Bernardo L Horta, Fernando Hartwig, Gabriel Victora, Lúcia C Pellanda, Odir A Dellagostin, Claudio J Struchiner, Marcelo N Burattini, Marcelo R Gonçalves, Lia G Possuelo, Liliana P Weber, Sonara Lucia Estima, Nadège Jacques, Jenifer Härter, Shana G Silva, Matias Frizzo, Rosangela C Lima, Fernando C Barros, Mariângela F Silveira, Pedro C Hallal

**Affiliations:** I Brasil Universidade Federal de Pelotas. Centro de Pesquisas Epidemiológicas. Pelotas, RS, Brasil; II USA The Rockefeller University. New York, NY, USA; III Brasil Fundação Universidade Federal de Ciências de Saúde de Porto Alegre. Porto Alegre, RS, Brasil; IV Brasil Universidade Federal de Pelotas. Centro de Desenvolvimento Tecnológico. Pelotas, RS, Brasil; V Brasil Fundação Getúlio Vargas. Escola de Matemática Aplicada. Rio de Janeiro, RJ, Brasil; VI Brasil Universidade de São Paulo. Faculdade de Medicina. São Paulo, SP, Brasil; VII Brasil Universidade Federal de São Paulo. Hospital São Paulo. São Paulo, SP, Brasil; VIII Brasil Universidade Federal do Rio Grande do Sul. Porto Alegre, RS, Brasil; IX Brasil Universidade de Santa Cruz do Sul. Santa Cruz do Sul, RS, Brasil; X Brasil Universidade de Caxias do Sul. Caxias do Sul, RS, Brasil; XI Brasil Universidade La Salle (Unilasalle). Canoas, RS, Brasil; XII Brasil Universidade Federal do Pampa. Uruguaiana, RS, Brasil; XIII Brasil Universidade Federal da Fronteira Sul. Passo Fundo, RS, Brasil; XIV Brasil Universidade Regional do Noroeste do Estado do Rio Grande do Sul (UNIJUÍ). Ijuí, RS, Brasil; XV Brasil Universidade Federal de Santa Maria. Santa Maria, RS, Brasil; XVI Brasil Universidade Católica de Pelotas. Faculdade de Medicina. Pelotas, RS, Brasil

**Keywords:** Infecções por Coronavirus, prevenção & controle, Conhecimentos, Atitudes e Prática em Saúde, Comportamentos de Risco à Saúde, Fatores Socioeconômicos, Distanciamento Social, Estudo de Base Populacional

## Abstract

**OBJETIVO:**

Descrever práticas de distanciamento social em nove municípios do Rio Grande do Sul por sexo, idade, escolaridade e cidade.

**MÉTODOS:**

Foram realizados dois estudos transversais sequenciais representativos da população urbana nos municípios de Canoas, Caxias do Sul, Ijuí, Passo Fundo, Pelotas, Porto Alegre, Santa Cruz do Sul, Santa Maria e Uruguaiana com o intuito de estimar a prevalência populacional de Covid-19. Foi aplicado questionário contendo três perguntas sobre distanciamento social, cujas práticas foram submetidas a análises descritivas por subgrupos. Os dados foram comparados por testes qui-quadrado.

**RESULTADOS:**

Em termos de grau de distanciamento social, 25,8% dos entrevistados relataram estar praticamente isolados e 41,1% indicam praticar bastante distanciamento. Relataram ficar em casa o tempo todo 20,1% dos entrevistados, e 44,5% informam que saem apenas para atividades essenciais. Mais da metade dos domicílios não recebe visitas de não moradores. O grupo que relatou menos distanciamento social foi o de adultos entre 20 e 59 anos, enquanto mais de 80% dos entrevistados com 60 anos ou mais relataram estar praticamente isolados ou fazendo bastante distanciamento. As mulheres relataram fazer mais distanciamento que os homens, e os grupos de maior escolaridade foram os que relataram sair diariamente para atividades regulares com mais frequência.

**CONCLUSÕES:**

Os grupos mais jovens e mais idosos estão mais protegidos pelo distanciamento social, mas há grupos bastante expostos, o que pode ser um limitador importante no controle da progressão da epidemia de Covid-19.

## INTRODUÇÃO

No Brasil, desde a declaração de pandemia de Covid-19 pela Organização Mundial da Saúde, em 11 de março de 2020, estados e municípios, apoiados pelo Ministério da Saúde, começaram a estabelecer, ainda que com algumas diferenças, políticas e estratégias de distanciamento social. Rapidamente a maior parte do país passou a adotar medidas restritivas de contato pessoal, recomendando que as pessoas fiquem em casa, fechando escolas, atividades e locais que geram aglomerações, como eventos esportivos e *shopping centers*, e regulando o funcionamento de estabelecimentos comerciais. Em geral, isso significou fechar boa parte do comércio, com exceção de supermercados, lojas de alimentos, farmácias e outros segmentos considerados essenciais^1^.

Desde o início da pandemia se tem evidenciado que o distanciamento social pode reduzir a transmissão do Sars-CoV-2, vírus causador da Covid-19. Um estudo em Hong Kong encontrou redução de 44% na taxa de replicação (R_t_) da epidemia após a implementação de medidas de distanciamento social, em especial o fechamento de escolas^2^. Uma metanálise de 29 estudos, sendo 25 de modelagem, também concluiu que essas medidas podem reduzir a propagação da Covid-19, principalmente se associadas a ações amplas como fechamento de escolas e restrição a viagens^3^. Outra metanálise, que estudou os efeitos da distância e do uso de máscara e proteção ocular sobre o risco de infecção viral, mostrou que o distanciamento físico reduz em cerca de 80% a probabilidade de infecção (IC95% risco relativo: 0,10-0,41). Distâncias acima de um metro já seriam efetivas, mas dois metros são a distância ideal, e o uso de máscara também se mostrou altamente efetivo^4^.

No Brasil, utilizando dados da empresa In Loco^a^, que oferece serviços de inteligência a partir de dados de localização, o efeito do distanciamento social sobre a transmissão da epidemia foi avaliado em três estudos. Um deles encontrou associação inversa entre distanciamento social e a disseminação da doença e associação positiva entre mobilidade aérea e disseminação. Clima e características socioeconômicas apresentaram associação fraca^5^. Outro estudo mostrou correlação negativa forte (r < –0,7) entre a proporção de pessoas ficando em casa e R_t_^6^. O índice de isolamento social calculado pela In Loco também foi utilizado em um modelo de elasticidade que mostrou que um índice 10% maior implicou em 26% menos casos de Covid-19, em média. Essa mesma variação se associou a 18% menos mortes^7^.

Contudo, as fontes de dados de mobilidade, como a In Loco e o Google^b^, não conseguem caracterizar subgrupos de uma população. Não é possível saber se quem fica em casa são adultos ou idosos, homens ou mulheres. Assim, usamos dados do estudo Epidemiologia da Covid-19 no Rio Grande do Sul (Epicovid19/RS)^c^ – cujo objetivo principal é estimar a prevalência populacional da infecção por Sars-CoV-2 – para apresentar os padrões de distanciamento social nas nove cidades gaúchas pesquisadas, avaliando diferenças por cidade, idade, sexo e escolaridade.

## MÉTODOS

O estudo está sendo realizado em nove municípios-sentinela do estado do Rio Grande do Sul, escolhidos por serem os maiores das regiões intermediárias do estado, definidas pelo Instituto Brasileiro de Geografia e Estatística (IBGE), mais o segundo maior município da região metropolitana de Porto Alegre. Os municípios escolhidos no Epicovid19/RS foram Canoas, Caxias do Sul, Ijuí, Passo Fundo, Pelotas, Porto Alegre, Santa Cruz do Sul, Santa Maria e Uruguaiana.

Em cada um dos municípios foi selecionada amostra de 500 domicílios a partir de 50 setores censitários urbanos com probabilidade proporcional ao tamanho, sendo incluídos dez domicílios por setor. Os domicílios foram selecionados aleatoriamente com base em uma lista de endereços do IBGE na primeira rodada. Na segunda rodada, os participantes foram selecionados de forma sistemática, a partir de um pulo de nove domicílios a contar dos entrevistados na rodada anterior. Domicílios em que não havia ninguém no momento da entrevista ou em que houve recusa em participar foram substituídos pelo domicílio vizinho. Em cada local participante os moradores foram listados e um deles selecionado de forma aleatória para ser entrevistado e testado para Covid-19.

O estudo prevê a realização de quatro rodadas independentes, uma a cada duas semanas. A primeira e a segunda rodadas aconteceram entre 11 e 13 e entre 25 e 27 de abril de 2020, respectivamente. Foi utilizado teste sorológico rápido que verifica a presença de imunoglobulinas M (IgM) e G (IgG) com resultado em 15 minutos, validado pelo grupo de investigadores^8^. Outros detalhes da metodologia do estudo são apresentados em outra publicação^9^.

O questionário utilizado no estudo coleta informações sobre sexo, idade e escolaridade do entrevistado, escolaridade máxima do domicílio, distanciamento social praticado e sintomas relacionados a Covid-19. De especial interesse para este trabalho são as três perguntas sobre distanciamento social: 1) “Com relação ao distanciamento social que está sendo orientado pelas autoridades de saúde, ou seja, ficar em casa e evitar contato com outras pessoas, quanto você acha que está conseguindo fazer?”, sendo as possíveis respostas, lidas para o entrevistado, “muito pouco”, “pouco”, “mais ou menos”, “bastante”, “praticamente isolado de todo mundo”; 2) “Como tem sido a sua rotina de atividades?”, com as repostas “fica em casa o tempo todo”, “sai apenas para coisas essenciais, como comprar comida”, “sai de vez em quando para fazer compras e esticar as pernas”, “sai todos os dias para alguma atividade”, “sai todos os dias, o dia todo, para trabalhar ou outra atividade regular”; e 3) “Pensando na rotina da casa, quem tem entrado na casa?”, com as respostas “somente os familiares que moram junto, se tiver, e mais ninguém”, “alguns parentes próximos visitam de uma a duas vezes por semana”, “alguns parentes próximos visitam quase que todos os dias”, “amigos, parentes ou outros visitam de uma a duas vezes por semana”, e “amigos, parentes ou outros visitam quase todos os dias”. Nos casos em que o morador selecionado era criança menor de 12 anos ou idoso incapaz de responder, as perguntas foram direcionadas para um adulto responsável.

O trabalho de campo foi realizado pela empresa Instituto Pesquisas de Opinião^d^ com a colaboração de universidades localizadas nos municípios pesquisados. Os entrevistadores foram selecionados entre alunos de cursos da área de saúde das universidades parceiras e foram treinados a realizar o teste e aplicar o questionário. Todos os requisitos de biossegurança foram adotados para proteger entrevistadores e participantes.

O protocolo de pesquisa foi aprovado pela Comissão Nacional de Ética em Pesquisa (processo 30721520.7.1001.5313). Todos os entrevistados ou seus responsáveis assinaram termo de consentimento livre e esclarecido após receber informações sobre os objetivos e procedimentos da pesquisa.

As análises apresentadas são essencialmente descritivas, baseadas no cálculo de percentuais de grupos e gráficos de barras. Quando necessário, as frequências foram comparadas usando testes qui-quadrado.

## RESULTADOS

A Tabela 1 apresenta o número total e por município de entrevistas realizadas nas duas primeiras rodadas do Epicovid19/RS, nos dias 11–13 e 25–27 de abril, assim como a distribuição da amostra por sexo, idade e escolaridade. As cidades foram comparadas usando um teste qui-quadrado. Foram realizadas 8.611 entrevistas, com maior proporção de mulheres, quase 60% da amostra total. Observamos também que a distribuição etária difere expressivamente da distribuição populacional estimada para 2020 pelo IBGE^e^, na qual temos 12% para o grupo etário de 0 a 10 anos, 13% para o grupo de 11 a 19 anos, 30% para o de 20 a 39 anos, 26% para o de 40 a 59 anos e 19% para o grupo de 60 anos ou mais. Em nossa amostra há muito menos crianças e adolescentes e um excesso de idosos, provavelmente pela ausência de pessoas no domicílio na hora da entrevista e recusas para fazer o teste que envolve uma punção de polpa digital. Entre todos os domicílios abordados, 9% se recusaram a participar e 11% foram substituídos, na maioria das vezes por não haver ninguém em casa.

**Tabela 1:** Distribuição da amostra por município de residência, sexo, idade e escolaridade para as rodadas 1 e 2. Epicovid19/RS, abril de 2020.

	Canoas	Caxias do Sul	Ijuí	Passo Fundo	Pelotas	Porto Alegre	Santa Cruz do Sul	Santa Maria	Uruguaiana	Todas
N total	832	1.000	923	1.000	1.000	896	1.000	961	999	8.611
Sexo (p = 0112)										
Masculino	45,2	43,2	39,7	41,3	39,5	39,8	42,5	39,8	39,2	41,1
Feminino	54,8	56,8	60,4	58,7	60,5	60,2	57,5	60,3	60,8	58,9
Idade (p < 0,001)										
0–10	1,8	3,4	3,5	3,1	4,0	2,5	3,9	2,9	4,6	3,3
11–19	2,8	6,3	4,9	5,4	4,5	2,7	5,6	5,1	6,2	4,9
20–39	30,4	26,4	28,0	32,0	28,1	26,3	26,7	30,9	23,9	28,1
40–59	33,5	34,7	30,6	30,8	32,8	34,9	33,3	31,8	33,2	32,9
≥ 60	31,5	29,1	33,1	28,7	30,6	33,6	30,5	29,2	32,0	30,9
Escolaridade do respondente (p < 0,001)										
Fundamental (0–4 anos)	5,7	5,5	7,5	3,4	7,0	3,3	6,5	4,1	5,1	5,3
Fundamental (≥ 5 anos)	25,6	29,7	33,3	27,9	29,6	18,7	34,1	23,0	34,8	28,7
Médio	33,8	31,9	31,2	31,3	31,5	29,8	28,9	31,7	35,6	31,7
Superior incompleto	12,8	12,3	8,7	9,8	9,6	11,1	9,8	11,0	7,1	10,2
Superior completo ou pós-graduação	22,2	20,7	19,3	27,6	22,4	37,0	20,7	30,3	17,3	24,1

Nota: Valores p se referem à comparação entre municípios usando teste qui-quadrado.

Em termos de grau de distanciamento social, a resposta mais frequente foi “bastante”, com 41,1% (Tabela 2). A resposta menos frequente foi “muito pouco”, com 5,8%. Os que se disseram estar praticamente isolados foram 25,8%.

**Tabela 2:** Distribuição dos três indicadores de distanciamento social para as rodadas 1 e 2. Epicovid19/RS, abril de 2020.

	N	%	IC 95%	
Com relação ao distanciamento social, quanto está fazendo?				
Isolado	2.225	25,8	24,6	27,1
Bastante	3.538	41,1	39,8	42,3
Mais ou menos	1.698	19,7	18,8	20,7
Pouco	648	7,5	6,9	8,2
Muito pouco	502	5,8	5,3	6,4
Como tem sido a rotina de atividades?				
Fica em casa o tempo todo	1.727	20,1	19,1	21,1
Sai apenas para coisas essenciais como comprar comida	3.836	44,5	43,3	45,9
Sai de vez em quando para compras e esticar as pernas	894	10,4	9,6	11,2
Sai todos os dias para alguma atividade	485	5,6	5,1	6,2
Sai todos os dias, o dia todo, para trabalhar ou outra atividade regular	1669	19,4	18,3	20,5
Quem tem entrado na casa?				
Só os familiares que moram junto, se tiver, e mais ninguém	4.584	53,2	51,6	54,9
Alguns parentes próximos visitam 1 a 2 vezes por semana	2.583	30,0	28,7	31,3
Alguns parentes próximos visitam quase que todos os dias	631	7,3	6,6	8,1
Parentes ou outras pessoas visitam 1 a 2 vezes por semana	458	5,3	4,8	5,9
Parentes ou outras pessoas visitam quase todos os dias	355	4,1	3,6	4,7

Relataram ficar em casa o tempo todo 20,1% dos entrevistados, e a maioria respondeu que sai apenas para atividades essenciais (44,5%). O grupo que sai todos os dias foi 19,4% do total (Tabela 2), e suas características foram exploradas para melhor entendê-los. Predominam homens (54,9%, p < 0,001) e adultos (90,3% entre 20 e 59 anos, p < 0,001) entre aqueles que relataram sair todos os dias para trabalho ou atividade regular. Entre os que saem todos os dias, apenas 7,6% têm 60 anos ou mais, contra 36,5% dos que não saem todos os dias, predominando também indivíduos com escolaridade mais alta (p < 0,001) – 39,2% dos que saem todos os dias têm nível superior incompleto ou mais, comparados com 33,1% entre os que não saem todos os dias. Esses resultados são apresentados na Tabela 3.

**Tabela 3:** Características dos indivíduos que relataram sair de casa todos os dias, o dia todo, para atividades regulares. Epicovid19/RS, abril de 2020.

	Sai todos os dias	

	Sim (%)	Não (%)
Sexo (n = 8.611)		
Masculino	54,9	37,8
Feminino	45,1	62,2
Idade (n = 8.609)		
0–10	0,3	4,1
11–19	1,8	5,6
20–39	47,1	23,5
40–59	43,2	30,4
≥ 60	7,6	36,5
Escolaridade do respondente (n = 8.350)		
Fundamental (0–4 anos)	2,8	6,0
Fundamental (≥ 5 anos)	18,6	31,2
Médio	39,4	29,8
Superior incompleto	12,3	9,7
Superior completo ou pós-graduação	26,9	23,4

Sobre o movimento na casa, mais da metade dos entrevistados relatou não receber ninguém além dos próprios moradores, e menos de 10% relataram visitas de não parentes.

Analisando os indicadores de distanciamento social por município, idade, sexo e escolaridade, encontramos diferenças estatisticamente significativas em todos os casos. Os resultados são apresentados nas Figuras 1 a 4 e nas Tabelas 4 a 7 [Table t5002] [Table t6002] [Table t7002]. Em relação ao grau de distanciamento social, Porto Alegre e Santa Maria apresentam o melhor perfil, enquanto Uruguaiana e Ijuí apresentam situação menos favorável. Na rotina de atividades, não há grande diferença entre as cidades em relação ao percentual que relata ficar em casa, mas chama atenção um percentual mais alto de pessoas que passam o dia todo fora de casa em Ijuí e Passo Fundo. Canoas se destaca em termos de movimento na casa, com a maior proporção de só moradores e a menor de parentes e outros. Porto Alegre e Santa Maria figuram logo atrás, em boa situação.

**Figura 1:** Indicadores de distanciamento social por município pesquisado utilizando dados das rodadas 1 e 2. Epicovid19/RS, abril de 2020. Nota: Valores p < 0,001 para grau de distanciamento, rotina de atividades e movimento na casa.

**Tabela 4:** Indicadores de distanciamento social por município participante do estudo. Epicovid19/RS, abril de 2020.

	Canoas	Caxias do Sul	Ijuí	Passo Fundo	Pelotas	Porto Alegre	Sta Cruz do Sul	Santa Maria	Uruguaiana
Com relação ao distanciamento social, quanto está fazendo? (p < 0,001)									
Isolado	26,8	26,8	22,9	25,2	27,9	31,0	24,2	27,7	20,6
Bastante	42,7	44,8	39,5	39,7	39,2	40,1	38,7	42,8	42,4
Mais ou menos	15,5	18,2	21,5	20,1	19,3	17,6	22,6	19,4	22,5
Pouco	7,5	5,6	8,5	9,5	7,1	6,5	7,6	6,6	8,9
Muito pouco	7,6	4,6	7,7	5,5	6,5	4,8	6,9	3,6	5,5
Como tem sido a rotina de atividades? (p < 0,001)									
Fica em casa o tempo todo	19,8	22,7	20,5	18,4	20,5	18,5	19,1	20,2	20,6
Sai apenas para coisas essenciais como comprar comida	43,5	44,0	41,7	43,5	47,8	46,7	42	47,8	43,9
Sai de vez em quando para compras e esticar as pernas	10,9	9,3	8,0	8,8	9,5	15,5	9,9	10,4	11,5
Sai todos os dias para alguma atividade	6,6	5,8	4,2	3,8	4,6	6,8	8,0	4,7	6,3
Sai todos os dias, o dia todo, para trabalhar ou outra atividade regular	19,1	18,2	25,6	25,5	17,6	12,5	21	17,0	17,6
Quem tem entrado na casa? (p < 0,001)									
Só os familiares que moram junto, se tiver, e mais ninguém	65,0	47,4	52,3	48,7	51,8	63,0	47,5	59,5	47,1
Alguns parentes próximos visitam 1 a 2 vezes por semana	25,8	32,1	32,8	30,4	32,5	23,6	33,6	24,0	33,7
Alguns parentes próximos visitam quase que todos os dias	4,0	9,6	5,5	10,5	8,1	5,0	7,7	6,0	8,5
Parentes ou outras pessoas visitam 1 a 2 vezes por semana	3,0	6,2	4,8	6,2	4,4	5,5	6,1	5,8	5,5
Parentes ou outras pessoas visitam quase todos os dias	2,2	4,7	4,6	4,2	3,2	3,0	5,1	4,6	5,2

**Tabela 5 t5002:** Indicadores de distanciamento social por grupos de idade. Epicovid19/RS, abril de 2020.

	Idade (anos)				

	0–10	11–19	20–39	40–59	≥ 60
Com relação ao distanciamento social, quanto está fazendo? (p < 0,001)					
Isolado	42,9	33,3	16,6	17,6	40,0
Bastante	34,8	37,5	38,5	43,6	42,0
Mais ou menos	11,9	19,7	23,9	23,9	12,3
Pouco	6,6	5,5	11,4	8,5	3,4
Muito pouco	3,8	4,0	9,6	6,5	2,2
Como tem sido a rotina de atividades? (p < 0,001)					
Fica em casa o tempo todo	58,9	35,9	9,3	9,3	34,7
Sai apenas para coisas essenciais como comprar comida	24,4	34,4	41,8	47,2	48,0
Sai de vez em quando para compras e esticar as pernas	9,1	15,9	10,1	11,0	9,3
Sai todos os dias para alguma atividade	5,9	6,7	6,2	7,1	3,4
Sai todos os dias, o dia todo, para trabalhar ou outra atividade regular	1,7	7,1	32,6	25,5	4,7
Quem tem entrado na casa? (p < 0,001)					
Só os familiares que moram junto, se tiver, e mais ninguém	53,0	45,4	57,3	54,5	49,5
Alguns parentes próximos visitam 1 a 2 vezes por semana	30,3	34,7	27,3	28,3	33,5
Alguns parentes próximos visitam quase que todos os dias	7,32	7,6	6,2	6,8	8,9
Parentes ou outras pessoas visitam 1 a 2 vezes por semana	4,9	7,1	5,4	6,4	3,8
Parentes ou outras pessoas visitam quase todos os dias	4,5	5,2	3,8	4,0	4,4

**Tabela 6 t6002:** Indicadores de distanciamento social por sexo do entrevistado. Epicovid19/RS, abril de 2020.

	Sexo	
	Masculino	Feminino
Com relação ao distanciamento social, quanto está fazendo? (p < 0,001)		
Isolado	21,3	29,0
Bastante	39,0	42,5
Mais ou menos	22,6	17,7
Pouco	9,2	6,4
Muito pouco	8,0	4,3
Como tem sido a rotina de atividades? (p < 0,001)		
Fica em casa o tempo todo	13,7	24,5
Sai apenas para coisas essenciais como comprar comida	40,9	47,1
Sai de vez em quando para compras e esticar as pernas	11,5	9,6
Sai todos os dias para alguma atividade	8,0	4,0
Sai todos os dias, o dia todo, para trabalhar ou outra atividade regular	25,9	14,8
Quem tem entrado na casa? (p < 0,001)		
Só os familiares que moram junto, se tiver, e mais ninguém	54,6	52,3
Alguns parentes próximos visitam 1 a 2 vezes por semana	28,0	31,4
Alguns parentes próximos visitam quase que todos os dias	6,7	7,8
Parentes ou outras pessoas visitam 1 a 2 vezes por semana	6,1	4,8
Parentes ou outras pessoas visitam quase todos os dias	4,7	3,7

**Tabela 7 t7002:** Indicadores de distanciamento social por escolaridade. Epicovid19/RS, abril de 2020.

	Escolaridade do respondente				


	Fundamental (0–4 anos)	Fundamental (≥ 5 anos)	Médio	Superior incompleto	Superior ou pós
Com relação ao distanciamento social, quanto está fazendo? (p < 0,001)					
Isolado	35,9	36,8	17,0	5,8	4,5
Bastante	31,4	36,2	20,5	7,1	4,8
Mais ou menos	21,0	41,7	22,0	8,5	6,8
Pouco	20,5	44,0	20,3	7,2	8,0
Muito pouco	23,5	46,5	17,3	7,4	5,3
Como tem sido a rotina de atividades? (p < 0,001)					
Fica em casa o tempo todo	38,3	35,9	9,6	5,8	10,3
Sai apenas para coisas essenciais como comprar comida	29,5	42,2	10,3	5,2	12,9
Sai de vez em quando para compras e esticar as pernas	15,1	45,6	9,4	5,3	24,6
Sai todos os dias para alguma atividade	10,6	45,7	12,4	7,5	23,8
Sai todos os dias, o dia todo, para trabalhar ou outra atividade regular	11,9	48,6	11,3	6,0	22,1
Quem tem entrado na casa? (p < 0,001)					
Só os familiares que moram junto, se tiver, e mais ninguém	41,9	39,5	10,5	4,3	3,8
Alguns parentes próximos visitam 1 a 2 vezes por semana	47,4	33,7	9,7	4,9	4,4
Alguns parentes próximos visitam quase que todos os dias	53,4	30,4	6,8	5,6	3,9
Parentes ou outras pessoas visitam 1 a 2 vezes por semana	56,6	28,3	5,3	5,6	4,2
Parentes ou outras pessoas visitam quase todos os dias	62,1	23,5	4,8	5,7	3,9

Os padrões de distanciamento social e rotina de atividades apresentam uma forma em U bem clara em relação à idade (Figura 2), ou seja, os grupos de 20 a 59 anos relatam menos estar praticamente isolados ou ficar em casa o dia todo. Nota-se nessa faixa etária frequência considerável de sair o dia todo, todos os dias. A faixa etária de 60 anos ou mais parece bastante protegida, com mais de 80% relatando estar praticamente isolada ou fazer bastante distanciamento e ficar em casa o dia todo ou sair só para coisas essenciais. O movimento da casa tem relação menos clara com a idade do respondente, o que é esperado, visto que dependente muito menos do respondente em si.

**Figura 2:** Indicadores de distanciamento social por grupo de idade do respondente utilizando dados das rodadas 1 e 2. Epicovid19/RS, abril de 2020. Nota: Valores p < 0,001 para grau de distanciamento, rotina de atividades e movimento na casa.

Em relação ao sexo (Figura 3), fica claro que as mulheres apresentam padrão mais favorável de distanciamento social e de rotina de atividades, com maior proporção de mulheres praticamente isoladas e ficando em casa o tempo todo. O movimento na casa pouco difere, apesar da significância estatística, como observado para idade.

**Figura 3:** Indicadores de distanciamento social por sexo do respondente utilizando dados das rodadas 1 e 2. Epicovid19/RS, abril de 2020. Nota: Valores p < 0,001 para grau de distanciamento, rotina de atividades e movimento na casa.

A escolaridade do respondente tem clara associação com o distanciamento social e a rotina de atividades (Figura 4). Se avaliamos o grupo que relatou estar praticamente isolado ou bastante distanciado, observamos um padrão em U, mas menos marcado do que para idade. Os indivíduos com ensino médio e superior incompleto são os que menos aderiram ao distanciamento social. Em contraste, na rotina de atividades os indivíduos com maior escolaridade tendem a relatar menos a permanência em casa. Na outra ponta da classificação, os grupos de ensino médio para cima são os que mais relatam sair diariamente para atividades regulares de trabalho. Restringir essas análises a adultos e idosos de forma a retirar indivíduos fora da faixa etária potencialmente ocupada não muda os padrões observados. Finalmente, temos associação clara entre aumento do percentual de domicílios com movimento exclusivo de familiares e maior escolaridade, ou seja, os indivíduos com maior escolaridade saem mais de casa, mas restringem mais a presença de não familiares.

**Figura 4:** Indicadores de distanciamento social por escolaridade do respondente utilizando dados das rodadas 1 e 2. Valores p para grau de distanciamento, rotina de atividades e movimento na casa todos p < 0.001. Epicovid19/RS, abril de 2020.

## DISCUSSÃO

Este estudo oferece uma perspectiva do distanciamento social em nove municípios do Rio Grande do Sul e seus padrões por características sociodemográficas. O contexto do estado é de baixa prevalência de Covid-19, como mostraram as duas primeiras rodadas do Epicovid19/RS, com estimativas de 0,05% e 0,13%, respectivamente^10^. Entre a primeira e a segunda rodadas da pesquisa iniciou-se no estado um processo de reabertura controlada de alguns segmentos do comércio, cujo efeito sobre os indicadores de distanciamento social deve ser sentido de forma mais evidente a partir da terceira rodada. Do ponto de vista dos padrões de distanciamento, não houve mudança relevante da primeira para a segunda rodada, de forma que julgamos melhor usar os dados conjuntamente.

As informações sobre distanciamento e mobilidade se baseiam em relatos e podem estar sujeitas a vieses de memória ou, mais importante, de expectativa social. No atual momento, o respondente pode ser sentir constrangido em revelar fraca aderência às práticas preconizadas para seu município. Nossos resultados, nesse sentido, podem superestimar o distanciamento social. Outra limitação dos dados refere-se à amostra não ser representativa do estado, mas baseada em um conjunto selecionado de cidades. Ainda assim, acreditamos que o quadro apresentado nos oferece um perfil informativo e útil do comportamento da população nas cidades estudadas, que congregam parcela significativa dos habitantes do estado.

Pelo relatado, cerca de 65% dos entrevistados aderiram ao distanciamento social e mais de 80% dos domicílios restringiram as visitas a moradores ou parentes próximos até duas vezes por semana. Observa-se também que há variações expressivas entre cidades, apesar de serem todas do mesmo estado. Duas das três cidades com maior percentual de acesso apenas aos moradores situam-se na região metropolitana, mas a terceira é uma cidade de menor porte, localizada no centro do estado. Não se identifica de forma clara um padrão relacionado a porte ou localização geográfica.

Em relação a idade e escolaridade, vemos que crianças, adolescentes e idosos estão mais protegidos em termos de distanciamento social, estando os adultos de 20 a 59 anos mais expostos. Esse é um resultado importante, visto o consenso já existente na literatura sobre idosos e portadores de comorbidades serem os grupos de maior risco para quadros graves e óbito^11,12^.

Os mais escolarizados aparecem menos protegidos em nossa análise, dado que evidentemente o grupo que relata sair de casa todos os dias para atividades regulares é composto essencialmente por adultos de escolaridade mais alta, predominando homens. Essa constatação surpreende, visto que se esperaria que este perfil profissional estivesse trabalhando a distância, em escritório doméstico, e que o trabalhador manual, da construção ou indústria, estivesse quotidianamente saindo para atividades laborais. Talvez a drástica redução das atividades econômicas possa explicar o achado. Ao mesmo tempo, as casas de pessoas com maior escolaridade relatam menos circulação de não moradores.

Em conclusão, vemos que os padrões de distanciamento social variam de forma significativa entre as cidades estudadas e entre subgrupos com diferentes características sociodemográficas. Os idosos, um dos principais grupos de risco, apresentam os melhores indicadores de distanciamento, assim como as crianças e os adolescentes, enquanto adultos com maior nível de escolaridade compõem o grupo com menor adesão ao distanciamento social.

Com a realização das rodadas seguintes do estudo no estado do Rio Grande do Sul será possível avaliar as mudanças de comportamento que podem ter ocorrido a partir da flexibilização das medidas restritivas, desde o final de abril de 2020.

## Footnotes

a Inloco. Enfrentando a COVID19. São Paulo; c2020 [citado 26 maio 2020]. Disponível em: http://www.inloco.com.br/covid-19

b Google. COVID-19: relatórios de mobilidade da comunidade. [citado 26 maio 2020]. Disponível em: https://www.google.com/covid19/mobility/

c Hallal PC, Horta BL, Barros AJD, Dellagostin AO, Hartwig FP, Pellanda LC, et al. Epidemiologia da COVID19 no Rio Grande do Sul: estudo de base populacional. Pelotas, RS: EPICOVID19; c2020 [citado 26 maio 2020]. Disponível em: http://www.rs.epicovid19brasil.org

d Instituto Pesquisas de Opinião. Porto Alegre, RS: IPO; c2016 [citado 26 maio 2020]. Disponível em: https://www.ipo.inf.br/

e Instituto Brasileiro de Geografia e Estatística. Projeções da População. Rio de Janeiro: IBGE; 2018 [citado 26 maio 2020]. Disponível em: https://www.ibge.gov.br/estatisticas/sociais/populacao/9109-projecao-da-populacao.html?=&t=resultados
